# Author Correction: Plant LHC-like proteins show robust folding and static non-photochemical quenching

**DOI:** 10.1038/s41467-022-29099-6

**Published:** 2022-03-16

**Authors:** Petra Skotnicová, Hristina Staleva-Musto, Valentyna Kuznetsova, David Bína, Minna M. Konert, Shan Lu, Tomáš Polívka, Roman Sobotka

**Affiliations:** 1 Institute of Microbiology, Academy of Sciences of the Czech Republic, Třeboň, Czech Republic; 2 Faculty of Science, University of South Bohemia, České Budějovice, Czech Republic; 3 Biology Centre, Institute of Plant Molecular Biology, Academy of Sciences of the Czech Republic, České Budějovice, Czech Republic; 4 School of Life Sciences, Nanjing University, Nanjing, China

**Keywords:** Non-photochemical quenching, Antenna complex, Protein folding

Correction to: *Nature Communications* 10.1038/s41467-021-27155-1, published 25 November 2021.

The original version of this Article omitted references to previous work by Mork-Jansson et al. and Mork-Jansson and Eichacker describing the heterologous expression of LIL3 in *E. coli*, LIL3 binding to chlorophyll *a* and LIL3 dimerization. This work should have been cited at the beginning of the fourth paragraph of the Introduction.

The original sentence stated “Although LIL3 and ELIP proteins probably bind Chl molecules in vivo^17,18^, how the associated pigments are organized and what exactly is the mechanism of binding are long-standing questions”. The corrected version reads “Although LIL3 and ELIP proteins probably bind Chlmolecules in vivo^17,18^ and the binding of Chl to LIL3 in vitro has been reported^19,20^, how the associated pigments are organized and what exactly is the mechanism of binding are long-standing questions”.

These references have been added as reference numbers [19, 20].

In addition, the fourth sentence of the Abstract previously stated “Whether the LHC-like proteins bind pigments has remained unclear”. The corrected version reads “How pigments associated with LHC-like proteins are organised and how they contribute to protein function has not yet been determined”

Mork-Jansson, A. E. et al. Lil3 dimerization and chlorophyll binding in *Arabidopsis thaliana*. *FEBS Lett.* **589**, 3064–3070 (2015).

Mork-Jansson, A. E. & Eichacker, L. A. Characterization of chlorophyll binding to LIL3. *PLoS ONE* **13**, e0192228 (2018).

This has been corrected in the PDF and HTML versions of the Article.

