# Supplementary material for: Plant LHC-like proteins show robust folding and static non-photochemical quenching
Source: Nat Commun. 2021 Nov 25;12:6890. doi: 10.1038/s41467-021-27155-1 (PMC8617258; doi:10.1038/s41467-021-27155-1)
Supplement: Supplementary file 1 — Supplementary Information [file 41467_2021_27155_MOESM1_ESM.pdf]

SUPPLEMENTARY FIGURES

Plant LHC-like proteins show robust folding and static non-photochemical quenching

His-LIL3.1 (LIL3)

MHHHHHHHHASSDSGSTSPTAAVSVEAPEPVEVIVKEPPQSTPAVKKEETATAKNVAVEGEEMKTTESVV
KFQDARWINGTWDLKQFEKDGKTDWDSVIVAEAKRRKWLEENPETTSNDEPVLFDTSIIPWWAWIKRYHL
PEA**E**LL**N**G**R**AAMIGFFMAYFVDSLTGVGLVDQMGNFFCKTLLFVAVAGVLFIRKNEDVDKLKNLFDETTL
YDKQWQAAWKNDDDESLGSKKK

His-ELIP2 (ELIP2)

MHHHHHHHHAQGDPIKEDPSVPSTSTSATPPQMPQSPPPPVSKPKVSTKFGDLLAFSGPAP**E**RI**N**G**R**LAM
VGFVAAIAMELSKGENVFAQISDGGVGWFLGTTALLTLASMVPLFKGIRAEAKSKGFMTSDA**E**LW**N**G**R**FA
MLGLVALAFTEYVTGGTLV

His-Li-ELIP (Li-ELIP)

MHHHHHHHHASSDSGSTSPTAAVSVEAPEPVEVIVKEPPQSTPAVKKEETATAKNVAVEGEEMKTTESVV
KFQDARWINGTWDLKQFEKDGKTDWDSVIVAEAKRRKWLEENPETTSNDEPVLFDTSIIPWWAWIKRYHL
PEA**E**RI**N**G**R**LAMVGFVAAIAMELSKGENVFAQISDGGVGWFLGTTALLTLASMVPLFKGIRAEAKSKGFM
TSDA**E**LW**N**G**R**FAMLGLVALAFTEYVTGGTLV

Supplementary Fig. 1. **Amino-acid sequences of LIL3, ELIP2, and Li-ELIP proteins expressed in *Synechocystis*.** 8xHis-tag is highlighted in red and the LIL3 N-terminus in the chimeric Li-ELIP protein in blue. LHC-like transmembrane helices are indicated with orange boxes, the Chl-binding motif ExxNxR is in bold. The second transmembrane helix is indicated by a green box. The predicted signal peptides of LIL3 (residues 1-39) and ELIP2 (residues 1-43) were removed.

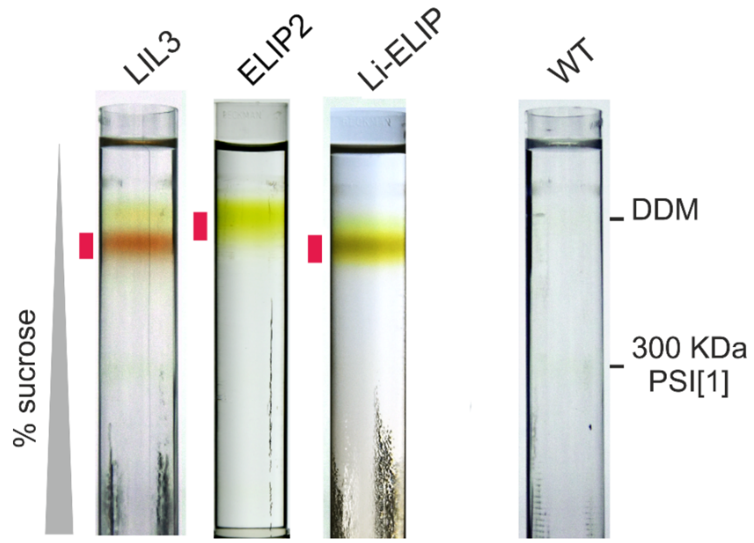

Supplementary Fig. 2. Sucrose gradient of isolated LIL3, ELIP2, and Li-ELIP proteins. His-tagged LIL3, ELIP2 and Li-ELIP proteins were purified from *Synechocystis LIL3*, *ELIP2* and *Li-ELIP* strains on a nickel column and further separated on a sucrose gradient (see Methods). Solubilized membranes from *Synechocystis* wild type (WT) cells were loaded on a nickel column as a purity control. Fractions marked by red boxes were collected; DDM indicates dodecyl-β-maltoside micelles (~ 70 KDa)[4], PSI[1] marks monomeric PSI.

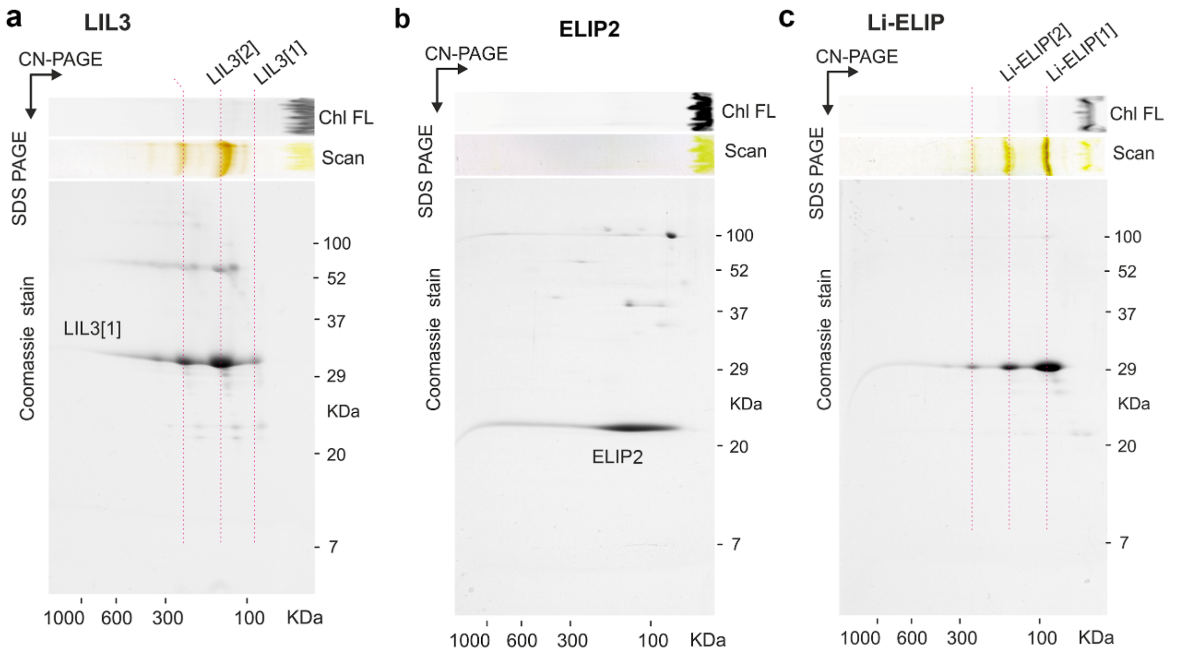

Supplementary Fig. 3. **2D CN/SDS-PAGE of the purified LIL3, ELIP2, and Li-ELIP proteins. a** Purified LIL3 (1.4 µg of Chl) was separated with CN-PAGE. The gel was photographed (Scan) and Chl fluorescence (Chl FL) detected after excitation with blue light. Proteins were further separated by SDS-PAGE in the second dimension and the 2D gel was stained with Coomassie Brilliant Blue. LIL3[1] and LIL3[2] indicate monomeric and dimeric LIL3, respectively. An identical analysis as described in (**a**) for the ELIPs (**b**) and Li-ELIP (**c**); a volume of elution corresponding to 1.4 µg of Chl was loaded for each protein.

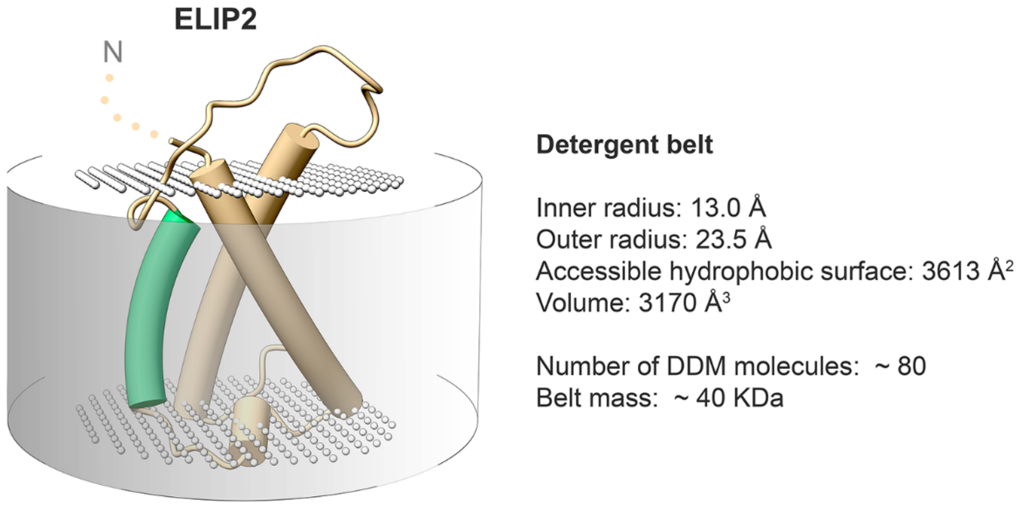

Supplementary Fig. 4. Predicted mass of the detergent belt for the structural model of ELIP2. The structural model of ELIP2 was prepared using iTASSER[5]. The accessible hydrophobic surface of the ELIP2 has been calculated using Det.Belt server (https://detbelt.ibcp.fr/)[6] and the number of detergent molecules estimated according to ref[6]. The predicted DDM detergent belt surrounding the ELIP2 protein is visualized as a grey cylinder.

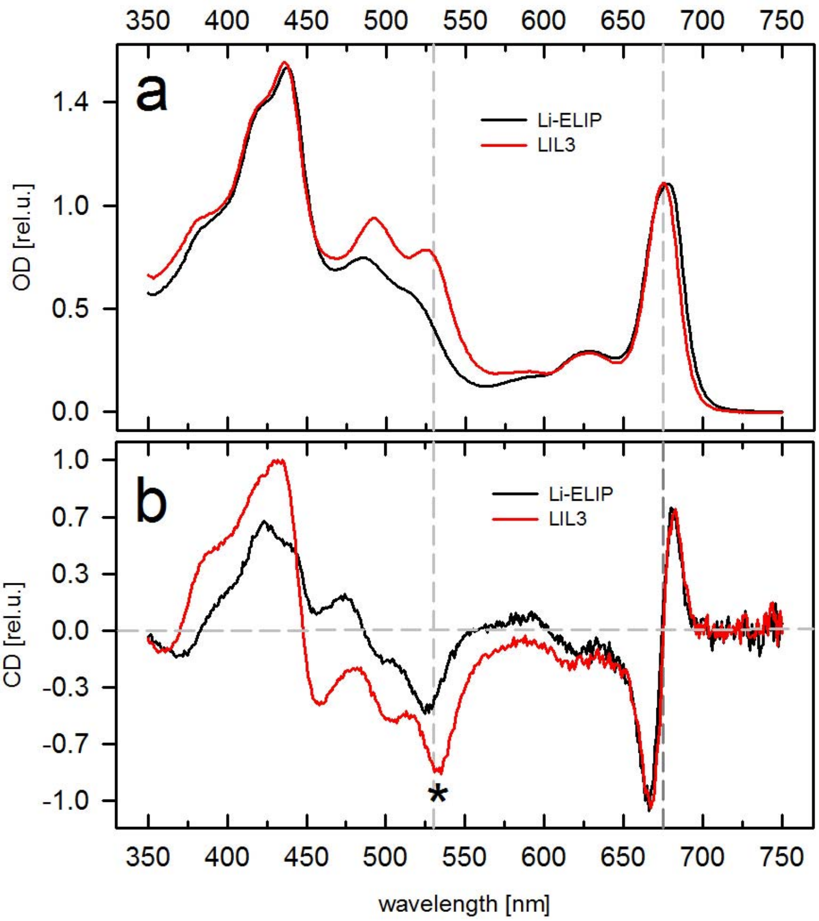

Supplementary Fig. 5. Comparison of absorption (**a**) and circular dichroism (**b**) spectra of LIL3 and Li-ELIP complexes recorded in the visible range. To facilitate comparison, spectra were normalized to maxima in Chl Q_y bands. Vertical dashed lines are placed at 530 and 675 nm. Asterisk highlights the main carotenoid CD band around 530 nm, for detail see main text.

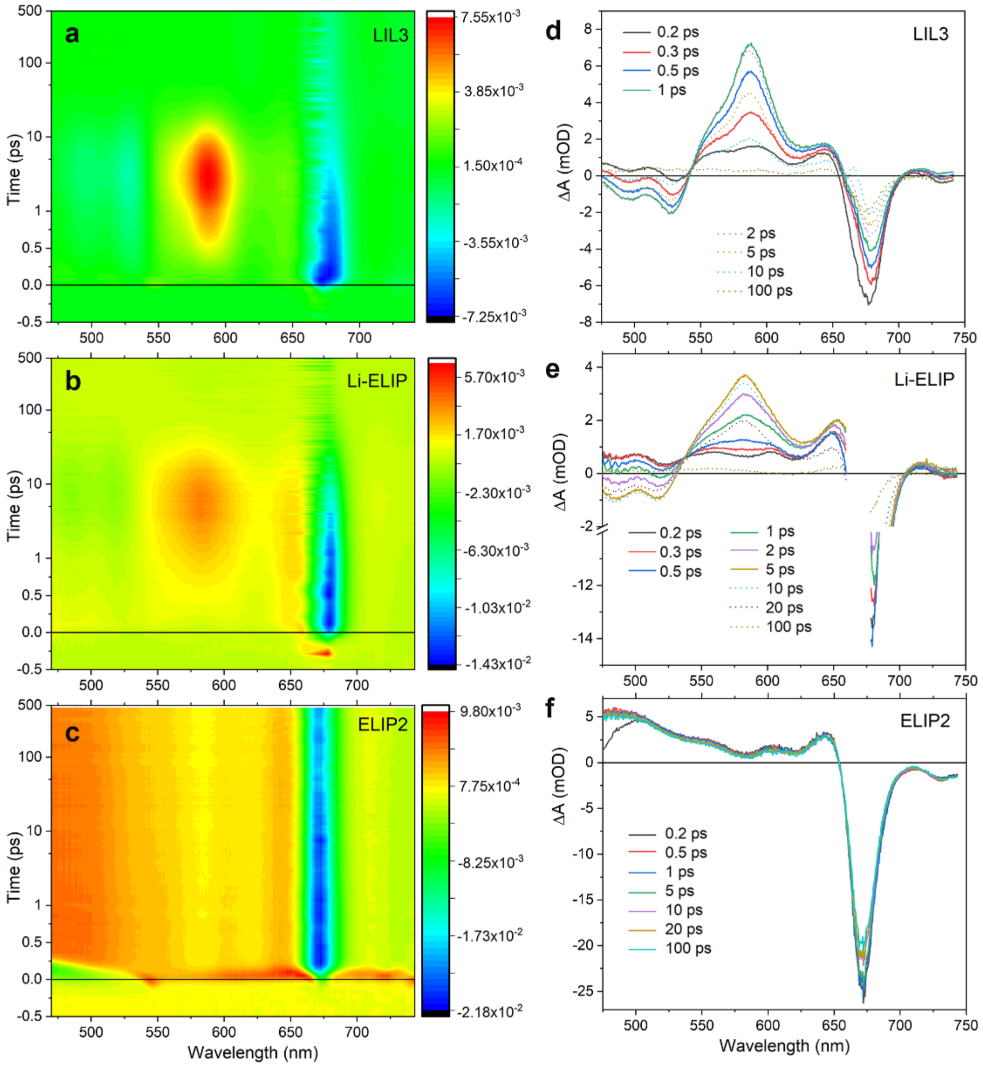

Supplementary Fig. 6. Ultrafast transient absorption data presented as 3D plots (**a-c**), and transient absorption spectra at selected delay times (**d-f**) measured for LIL3 (top row; **a**,**d**), Li-ELIP (middle row; **b**,**e**), and ELIP2 (bottom row; **c**,**f**).

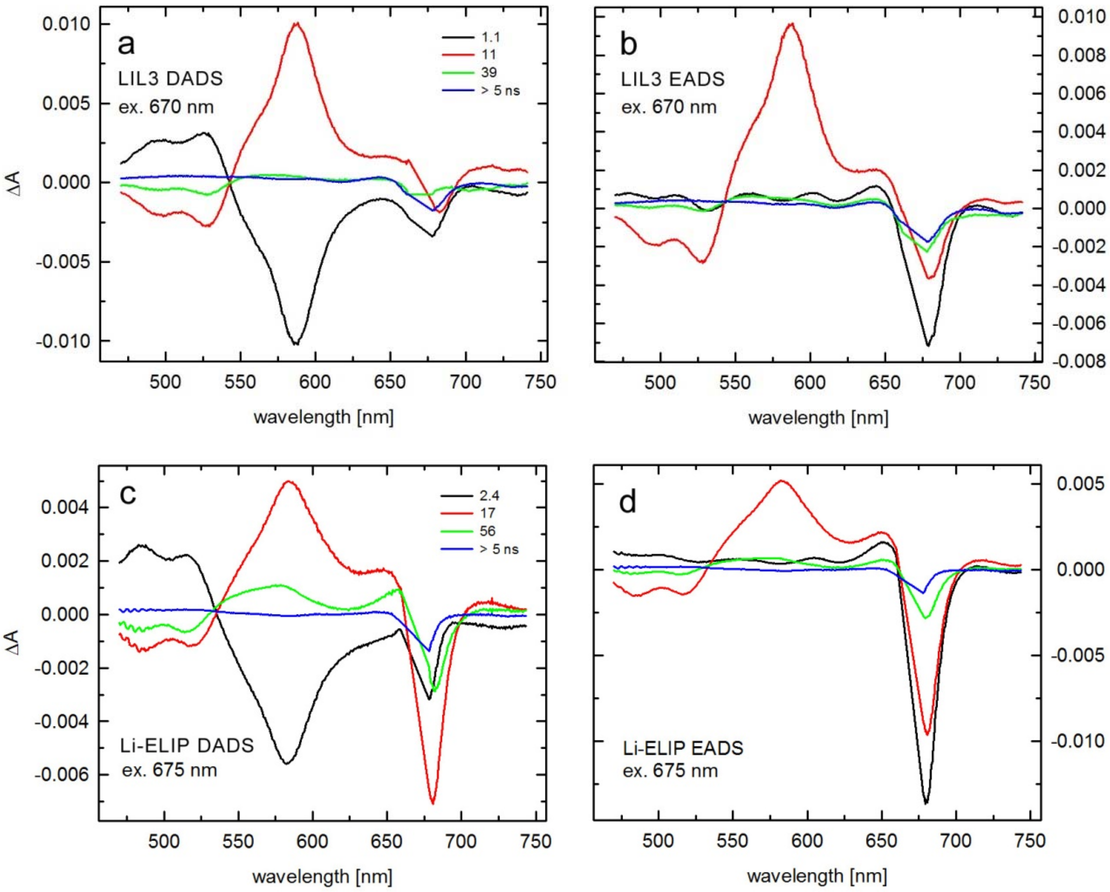

Supplementary Fig. 7. Global analysis of LIL3 and Li-ELIP transient absorption data, after excitation into the Chl Q_y band. **a**,**b** Decay-Associated Difference Spectra (DADS) and Evolution-Associated Difference Spectra (EADS) of LIL3. **c**,**d** DAS and EADS of Li-ELIP. The respective time constants are given in the legend. Note that the region ±6 nm around the pump wavelength was omitted from the analysis.

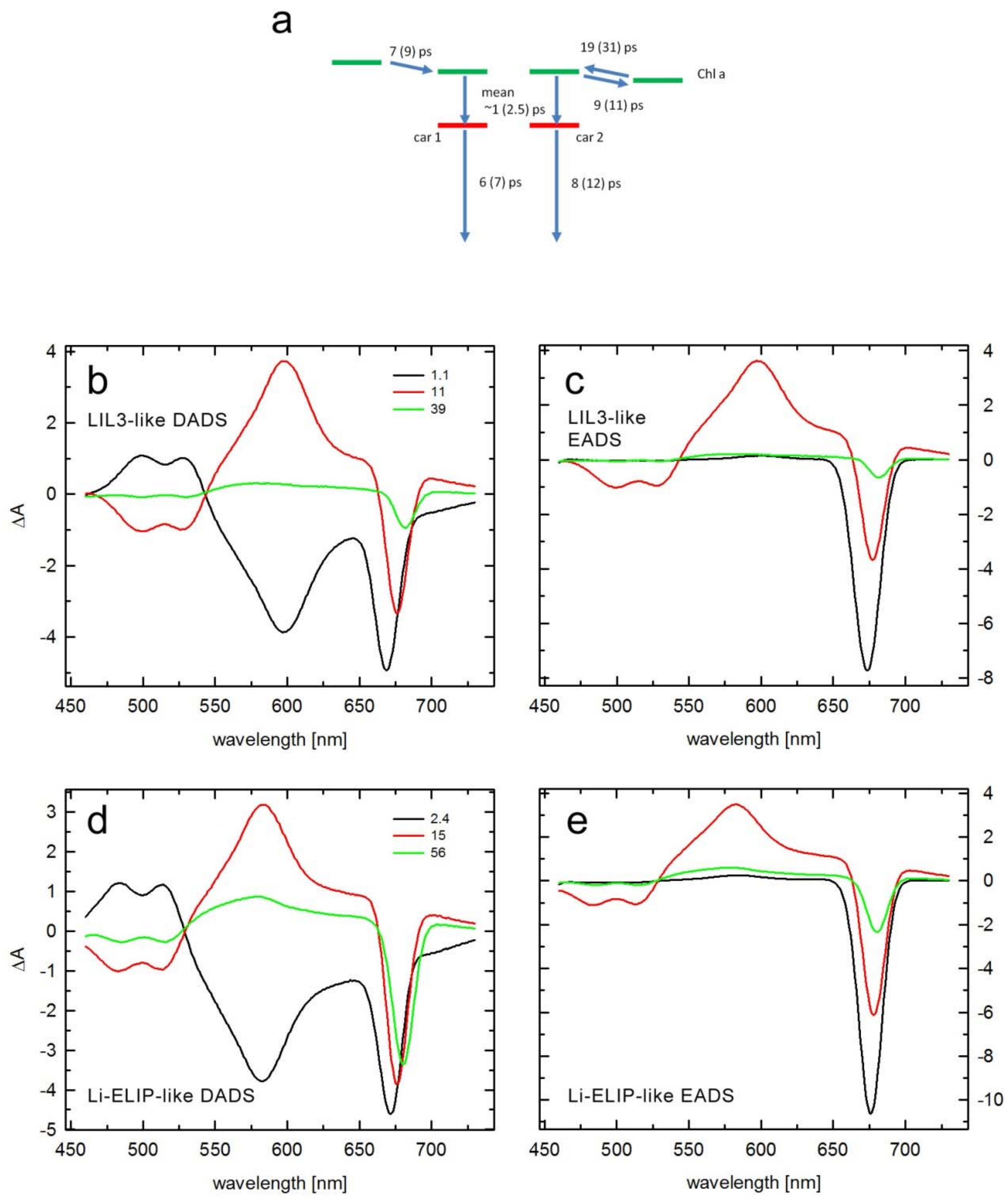

Supplementary Fig. 8. Simulation of LIL3 and Li-ELIP excited state dynamics based on unified reaction scheme. **a** The analysis was based on the following observations:

i) there are at least 3-4 Chl compartments and two carotenoid compartments forming two energy transfer pathways

ii) both the steady-state spectroscopy, especially CD, and the time-resolved spectroscopy suggest that the functioning of the LIL3 and Li-ELIP protein is similar

iii) the lifetimes of carotenoid states are unlikely to be longer than 15 ps, considering the published lifetimes of free and LHC-bound carotenoids[7,8]. Consequently, the

observed decay components in the tens-of-picoseconds range must be due to energy equilibration steps

iv) all components shown in Supplementary Fig. 6 are mixture of carotenoid and chlorophyll dynamics, because the global analysis is not capable of extracting a pure decay of carotenoid S_1 state

v) excitation into Chl is not selective – all Chl states are excited.

Based on this, we have developed the model shown in Panel (**a**) for the excitation energy pathways in both LIL3 and Li-ELIP proteins and applied it to simulate the DADS/EADS obtained by directly fitting the data to a sequential model shown in Supplementary Fig. 6. The time constants used for simulation are shown in the scheme in Panel (**a**), the numbers in parentheses corresponds to Li-ELIP simulation. Note that the Chl component with the longest lifetime (> 5 ns) was not modelled.

b-e DADS and EADS modelled using the scheme given in (**a**). (**b**,**c**) – simulation of the LIL3 protein, (**d**,**e**) – simulation of Li-ELIP protein. These data were modelled using the spectra of 17-ps DADS of Li-ELIP and 56-ps DADS of LI-ELIP, scaled to identical bleaching at 0-1 vibronic transition. Each Chl compartment was modelled by a single Gaussian curve. Carotenoids of LIL3 and Li-ELIP were simulated using the same spectral shapes, only for LIL3 the spectra were shifted by 15 nm to the red compared to Li-ELIP. These data should be compared to Supplementary Fig. 6.

To achieve adequate simulation of the experimental data, the Chl pool was separated into four compartments composing a fast and slow part, where only two of Chl's couple directly to carotenoids, and are quenched with time constants in the ~ ps range. Detailed dynamics of these "fast" pigments is not properly resolved and they are gathered under a single fast component characterized with time constants of 1 ps (2.5 ps) for LIL3 (and Li-ELIP) that exhibit features indicative of Chl decay (negative Chl bands in DADS) and population of carotenoid S_1 states (negative carotenoid bands in DADS). Overall, the dynamics is determined by equilibration between these fast-decaying Chl states and the remainder of Chl pool. This is reflected in the slower DADS (EADS) that combine features characteristic of both carotenoid and Chl excited state decays. As a result of such dynamics, pure components corresponding to carotenoid excited state decay and their time constants cannot be resolved.

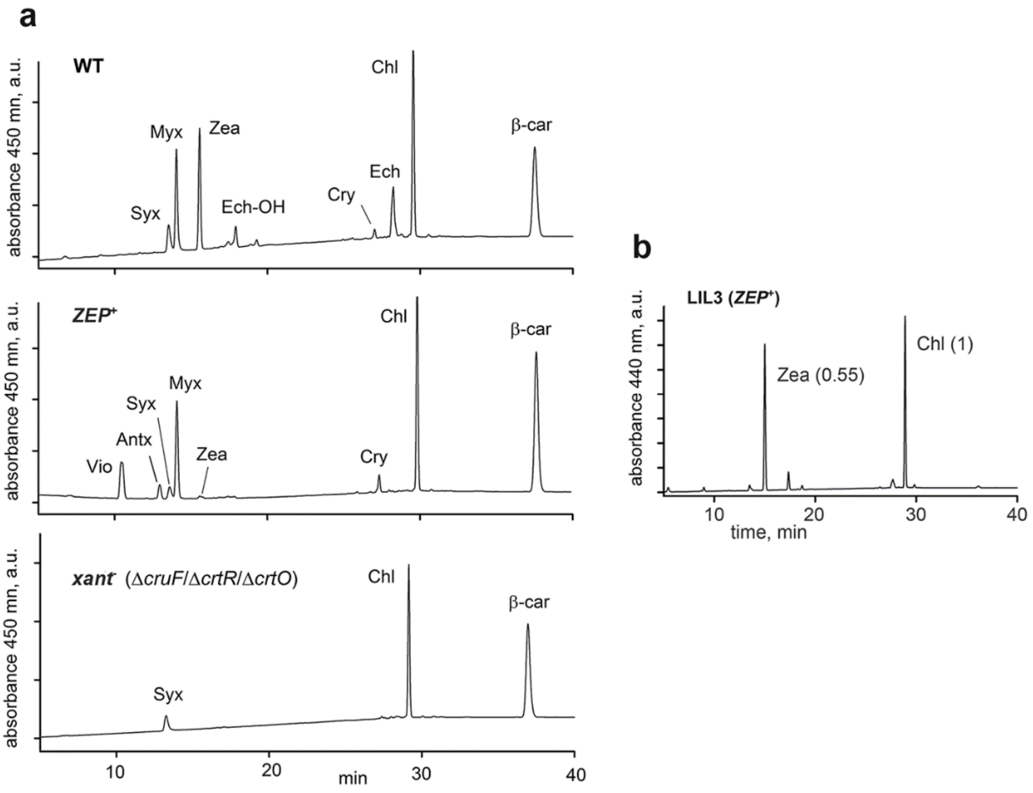

Supplementary Fig. 9. **Pigment profiles of *Synechocystis* WT, *ZEP*$^{+}$ and *xant*$^{-}$ strains. a** Pigments were extracted from cell pellets in 100% methanol and analysed by HPLC according to the protocol described in the Materials and Methods section. Syx – synechoxanthin; Myx – myxoxanthophyll; Zea – zeaxanthin; Ech-OH – hydroxy-echinenone; Cry – cryptoxanthin; Ech – echinenone; β-car – β-carotene; Vio – violaxanthin; Antx – antheraxanthin. **b** Pigments associated with LIL3 purified from the ZEP+ genetic background. Pigments were extracted from a ~ 20 times concentrated elution obtained from the nickel column and analysed by HPLC; molar stoichiometries of the identified pigments are shown in parentheses. Values represent means of three technical replicates, all standard deviations were below 10%.

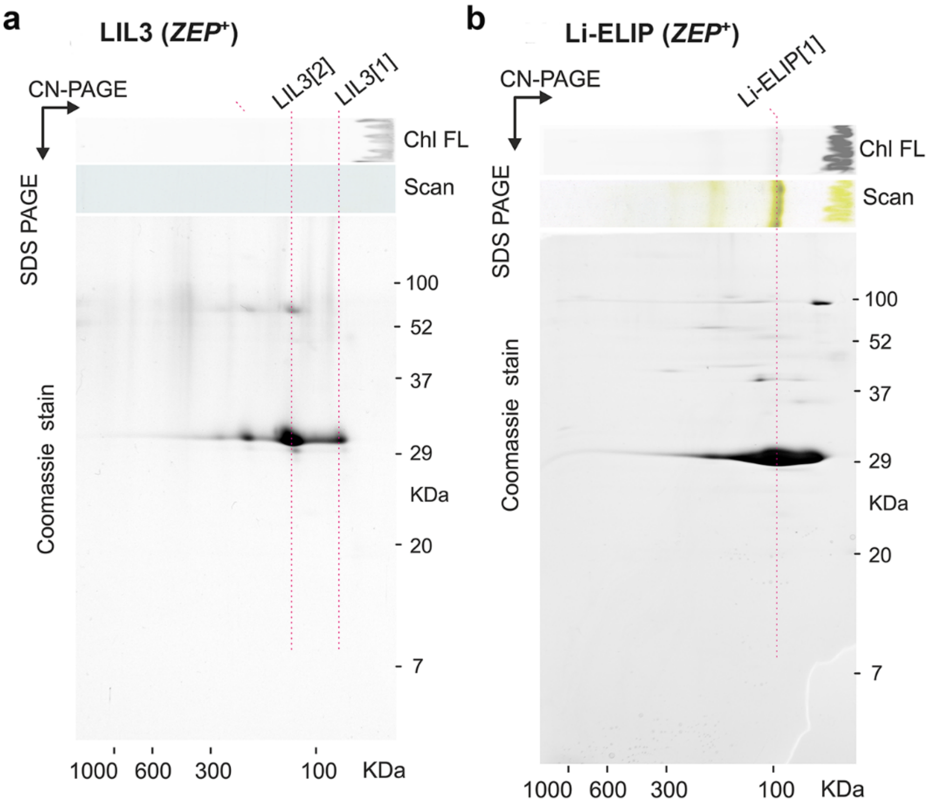

Supplementary Fig. 10. **2D CN/SDS-PAGE of the LIL3 and Li-ELIP proteins purified from the *ZEP*⁺ strain. a** The colourless LIL3 purified from the *ZEP*⁺ strain was separated with CN-PAGE; 1/5 of the total volume of the elution was loaded. The gel was scanned (Scan) and Chl fluorescence (Chl FL) detected after excitation with blue light. Proteins were further separated by SDS-PAGE in the second dimension and the 2D gel was stained with Coomassie Brilliant Blue. LIL3[1] and LIL3[2] indicate monomeric and dimeric LIL3, respectively. **b** An identical analysis as described in (**a**) for the Li-ELIP; 1.4 μg of Chl was loaded.

.

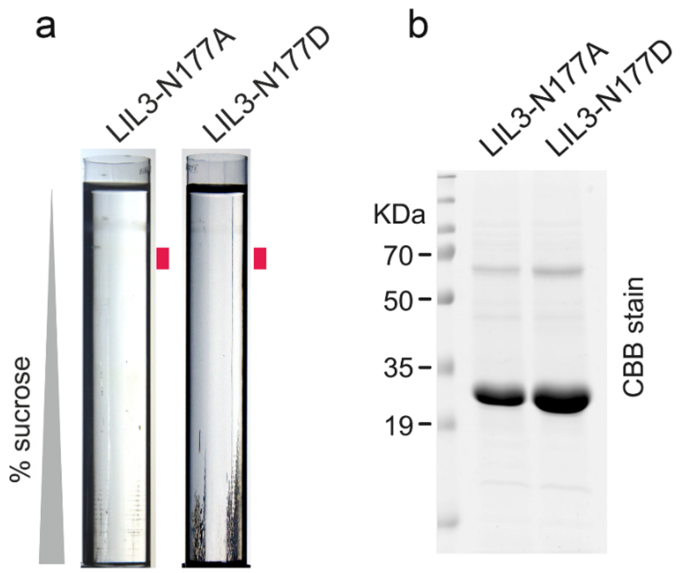

Supplementary Fig. 11. **Purification of mutated LIL3-N177A and LIL-N177D variants. a** Both proteins were isolated from *Synechocystis* using a nickel column. Eluted proteins were concentrated ~ 12 times and loaded on the top of the sucrose gradient. After ultracentrifugation, the fraction corresponding to the mobility of non-mutated (pigmented) LIL3 (see Supplementary Fig. 2) was collected for each mutated variant, concentrated again, and separated on SDS-PAGE (**b**). The gel was stained with Coomassie Brilliant Blue.

SUPPLEMENTARY TABLE

Plant LHC-like proteins show robust folding and static non-photochemical quenching

Supplementary Table 1. A list of *Synechocystis* mutant strains described in this study. WT – *Synechocystis* wild type (see Material and Methods for details). Km – kanamycin; Sp – spectinomycin; Ery – erythromycin.

strain	characteristics	source
LIL3	*8xHis-LIL3.1* (At4g17600) and Km^R replacement of *psbAII* in WT	this study
ELIP2	*8xHis-ELIP2* (At4g14690) and Km^R replacement of *psbAII* in WT	this study
Li-ELIP	*8xHis-Li-ELIP* (chimeric) and Ery^R replacement of *psbAII* in WT	this study
ZEP$^+$	zeaxanthin epoxidase (At5g67030) and Km^R replacement of *crtO* in WT	ref[1]
xant$^-$	*crtR*, *crtO* and *crtF* genes replaced by Km^R or Ery^R (*crtR*), Sp^R (*crtO*) and Cm^R (*crtF*)	ref[2,3]
LIL3/ZEP$^+$	*8xHis-LIL3.1* and Cm^R replacement of *psbAII* in *ZEP+*	this study
LIL3/xant$^-$	*8xHis-LIL3.1* and Km^R replacement of *psbAII* in *xant*$^-$ (Ery^R, Sp^R, Cm^R)	this study
Li-ELIP/ZEP$^+$	*8xHis-Li-ELIP* and Cm^R replacement of *psbAII* in *ZEP*$^+$	this study
Li-ELIP/xant$^-$	*8xHis-Li-ELIP* and Ery^R replacement of *psbAII* in *xant*$^-$ (Km^R, Sp^R, Cm^R)	this study
LIL3-N177A	*8xHis-LIL3.1-N177A* and Km^R replacement of *psbAII* in WT	this study
LIL3-N177D	*8xHis-LIL3.1-N177D* and Km^R replacement of *psbAII* in WT	this study

Supplementary Table 2. A list of primers used in this study.

Primer	Sequence
LIL3-His-NdeI	CTAGAGCATATGCATCATCATCATCATCATCATCATGCTTCCTCCGACAGTGGATCA
LIL3-BglII	GTCCTCAGATCTTTACTTCTTCTTAGAACCCAATG
ELIP2-His-NdeI	ATCGACATATGCATCATCATCATCATCATCATCATGCTCAGGGCGATCCTATCAA
ELIP2-BamHI	GTCTAGGATCCTTAGACTAGAGTCCCACCAG
LIL3-N177A_1	CCACTTACCTGAAGCTGAACTCTTAGCTGGTCGTGCGGCG
LIL3-N177A_2	CGCCGCACGACCAGCTAAGAGTTCAGCTTCAGGTAAGTGG
LIL3-N177D_1	CTGAAGCTGAACTCTTAGATGGTCGTGCGGCGA
LIL3-N177D_2	TCGCCGCACGACCATCTAAGAGTTCAGCTTCAG

SUPPLEMENTARY REFERENCES

Plant LHC-like proteins show robust folding and static non-photochemical quenching

1. Cao, T. J. *et al.* Manipulation of *Synechocystis* sp. PCC 6803 as a platform for functional identification of genes involved in carotenoid metabolism. *Plant Biotechnol J* **18**, 605-607 (2020).
2. Toth, T. N. *et al.* Carotenoids are essential for the assembly of cyanobacterial photosynthetic complexes. *Biochim. Biophys. Acta* **1847**, 1153-1165 (2015).
3. Proctor, M. S. *et al.* Xanthophyll carotenoids stabilise the association of cyanobacterial chlorophyll synthase with the LHC-like protein HliD. *Biochem. J.* **477**, 4021-4036 (2020).
4. Lipfert, J., Columbus, L., Chu, V. B., Lesley, S. A. & Doniach, S. Size and shape of detergent micelles determined by small-angle X-ray scattering. *J Phys Chem B* **111**, 12427-12438 (2007).
5. Yang, J. *et al.* The I-TASSER Suite: protein structure and function prediction. *Nat. Methods* **12**, 7-8 (2015).
6. Chaptal, V. *et al.* Quantification of detergents complexed with membrane proteins. *Sci Rep* **7**, 41751 (2017).
7. Staleva, H. *et al.* Ultrafast dynamics of long homologues of carotenoid zeaxanthin. *J Phys Chem A* **119**, 11304-11312 (2015).
8. Polívka, T. *et al.* Carotenoid S_1 state in a recombinant light-harvesting complex of Photosystem II. *Biochemistry* **41**, 439-450 (2002).
