# Supplementary material for: Plant LHC-like proteins show robust folding and static non-photochemical quenching
Source: Nat Commun. 2021 Nov 25;12:6890. doi: 10.1038/s41467-021-27155-1 (PMC8617258; doi:10.1038/s41467-021-27155-1)
Supplement: Supplementary file 3 — Reporting Summary [file 41467_2021_27155_MOESM3_ESM.pdf]

nature research

Corresponding author(s): Sobotka Roman

Last updated by author(s): Oct 18, 2021

Reporting Summary

Nature Research wishes to improve the reproducibility of the work that we publish. This form provides structure for consistency and transparency in reporting. For further information on Nature Research policies, see our Editorial Policies and the Editorial Policy Checklist.

Statistics

For all statistical analyses, confirm that the following items are present in the figure legend, table legend, main text, or Methods section.

n/a	Confirmed	
☐	☒	The exact sample size (*n*) for each experimental group/condition, given as a discrete number and unit of measurement
☐	☒	A statement on whether measurements were taken from distinct samples or whether the same sample was measured repeatedly
☐	☒	The statistical test(s) used AND whether they are one- or two-sided<br>*Only common tests should be described solely by name; describe more complex techniques in the Methods section.*
☒	☐	A description of all covariates tested
☒	☐	A description of any assumptions or corrections, such as tests of normality and adjustment for multiple comparisons
☒	☐	A full description of the statistical parameters including central tendency (e.g. means) or other basic estimates (e.g. regression coefficient) AND variation (e.g. standard deviation) or associated estimates of uncertainty (e.g. confidence intervals)
☒	☐	For null hypothesis testing, the test statistic (e.g. *F*, *t*, *r*) with confidence intervals, effect sizes, degrees of freedom and *P* value noted<br>*Give P values as exact values whenever suitable.*
☒	☐	For Bayesian analysis, information on the choice of priors and Markov chain Monte Carlo settings
☒	☐	For hierarchical and complex designs, identification of the appropriate level for tests and full reporting of outcomes
☒	☐	Estimates of effect sizes (e.g. Cohen's *d*, Pearson's *r*), indicating how they were calculated

Our web collection on statistics for biologists contains articles on many of the points above.

Software and code

Policy information about availability of computer code

Data collection	The transient absorption spectroscopy data were analyzed by freeware Glotaran (glotaran.org), modelling of excited-state dynamics was carried out with locally-written scripts in Matlab (code available upon reasonable request).
Data analysis	The following softwares or web servers were used for data analyses:<br>Det.Belt Server (https://detbelt.ibcp.fr); iTASSER (https://zhanglab.dcmb.med.umich.edu/I-TASSER/)

For manuscripts utilizing custom algorithms or software that are central to the research but not yet described in published literature, software must be made available to editors and reviewers. We strongly encourage code deposition in a community repository (e.g. GitHub). See the Nature Research guidelines for submitting code & software for further information.

Data

Policy information about availability of data

All manuscripts must include a data availability statement. This statement should provide the following information, where applicable:

- Accession codes, unique identifiers, or web links for publicly available datasets
- A list of figures that have associated raw data
- A description of any restrictions on data availability

The data that support the findings of this study are available from the corresponding author upon reasonable request.

Field-specific reporting

Please select the one below that is the best fit for your research. If you are not sure, read the appropriate sections before making your selection.

- [x] Life sciences
- [ ] Behavioural & social sciences
- [ ] Ecological, evolutionary & environmental sciences

For a reference copy of the document with all sections, see nature.com/documents/nr-reporting-summary-flat.pdf

Life sciences study design

All studies must disclose on these points even when the disclosure is negative.

Sample size	Proteins investigated in this study (LIL3, ELIP2 and Li-ELIP) were purified 3 times (see Replication field below). 2D gel separation, size-exclusion chromatography (SEC) and the analysis of fractions eluted from SEC was performed once for the each protein variant. 2D gel/ immunodetection of LIL3 in isolated membranes (Fig. 5b) was performed once. Ultrafast spectroscopy and CD spectroscopy was performed once for the purified LIL3, ELIP2 and Li-ELIP.
Data exclusions	No data exclusion
Replication	Proteins investigated in this study (LIL3, ELIP2 and Li-ELIP) were isolated repeatedly (each protein at least 3 times), which includes the mutated LIL3 variant. The quality and uniformity of all preparations was monitored spectroscopically (spectrum of pigments co-eluted with the protein) and by a HPLC pigment analysis of the crude elution. We did not observe any significant variability in pigment content among individual preparations. Pigment-less protein variants were compared only by 1D SDS-PAGE (yield, purity).<br><br>Ultrafast spectroscopy data point for each time delay was generated by averaging of 300 laser shots, the whole time profile consist of averaging 20 time scans, resulting in 6000 laser shots averaged for each data point.
Randomization	Due to the nature of the experimental setup, randomization was not applicable to the present study
Blinding	Due to the nature of the experimental setup, blinding was not applicable to the present study

Reporting for specific materials, systems and methods

We require information from authors about some types of materials, experimental systems and methods used in many studies. Here, indicate whether each material, system or method listed is relevant to your study. If you are not sure if a list item applies to your research, read the appropriate section before selecting a response.

Materials & experimental systems

n/a	Involved in the study	
[ ]	[x]	Antibodies
[x]	[ ]	Eukaryotic cell lines
[x]	[ ]	Palaeontology and archaeology
[x]	[ ]	Animals and other organisms
[x]	[ ]	Human research participants
[x]	[ ]	Clinical data
[x]	[ ]	Dual use research of concern

Methods

n/a	Involved in the study	
[x]	[ ]	ChIP-seq
[x]	[ ]	Flow cytometry
[x]	[ ]	MRI-based neuroimaging

Antibodies

Antibodies used	The primary antibodies used in this study were as follows: anti-LIL3.1 antibody (1 : 5000) raised in rabbit against the Thr-50 to Lys-84 recombinant fragment of the Arabidopsis protein; anti-ELIP2 antibody (1 : 5000) raised in rabbit again the synthetic peptide AQGD-PIKEDPSVPSC.<br><br>Secondary antibodies: goat anti-Rabbit IgG (whole molecule) Secondary Antibody, HRP (Sigma, catalogue number A6154)
Validation	anti-LIL3: (Lohscheider, J. N. et al. Altered levels of LIL3 isoforms in Arabidopsis lead to disturbed pigment-protein assembly and chlorophyll synthesis, chlorotic phenotype and impaired photosynthetic performance. Plant Cell Environ. 38, 2115-2127, 2015).<br><br>anti-ELIP2: (Sun, T. et al. ORANGE represses chloroplast biogenesis in etiolated Arabidopsis cotyledons via interaction with TCP14. Plant Cell 31, 2996-3014, 2019).
