# Supplementary material for: Plant LHC-like proteins show robust folding and static non-photochemical quenching
Source: Nat Commun. 2021 Nov 25;12:6890. doi: 10.1038/s41467-021-27155-1 (PMC8617258; doi:10.1038/s41467-021-27155-1)

Figure 1b

Full-sized (uncropped) gels are shown in Extended Data Fig. 3

Figure 1c

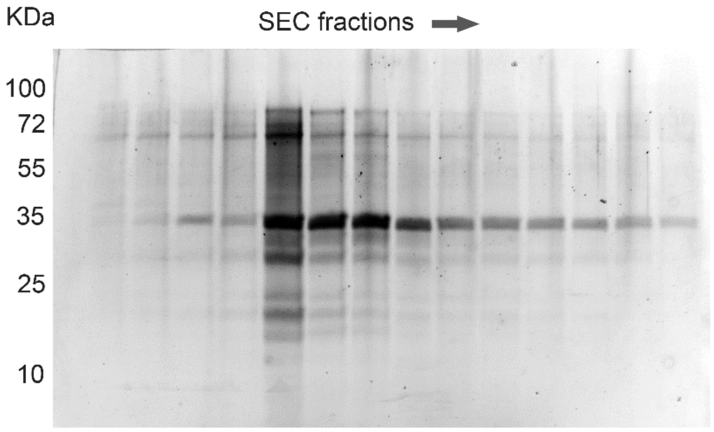

anti-LIL3

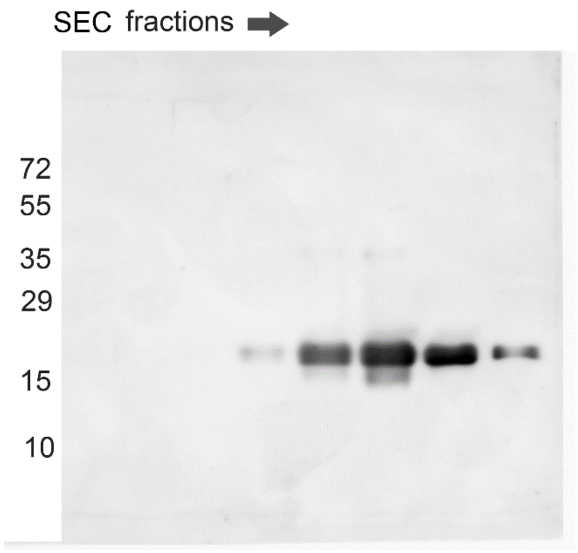

anti-ELIP2

Figure 2b

Full-sized (uncropped) gel) is shown in Extended Data Fig. 3

Figure 3, Extended Data Fig. 6

Attached excel file (Data_ultrafast_spectroscopy.xlsx) contains full spectro-temporal datasets obtained from ultrafast transient absorption measurements on LIL3, Li-ELIP and ELIP2. The first row is wavelength, the first column contains time points. 3D Plots in Extended Fig. 6 show these datasets after initial processing (chirp-correction). The file contains unprocessed raw data.

Figure 4a

Full-sized (uncropped) gels are shown in Extended Data Fig. 10

Figure 5a

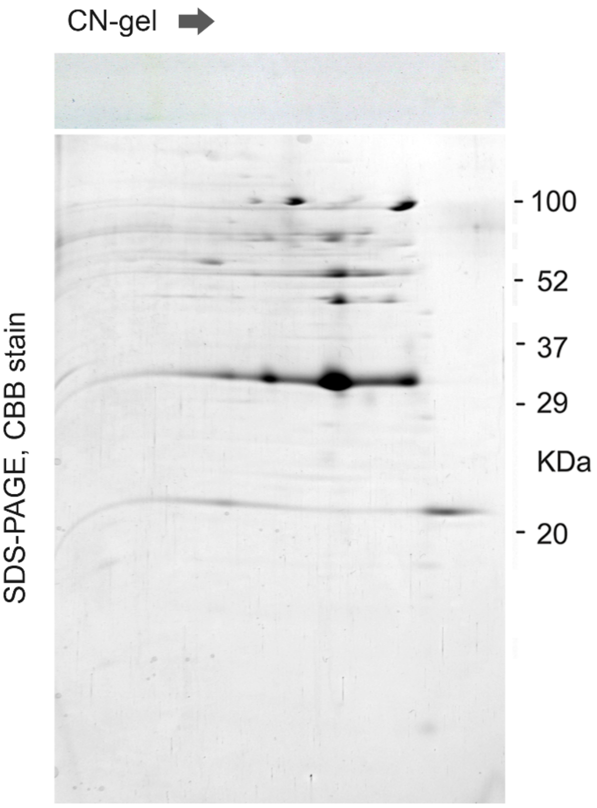

Figure 5b

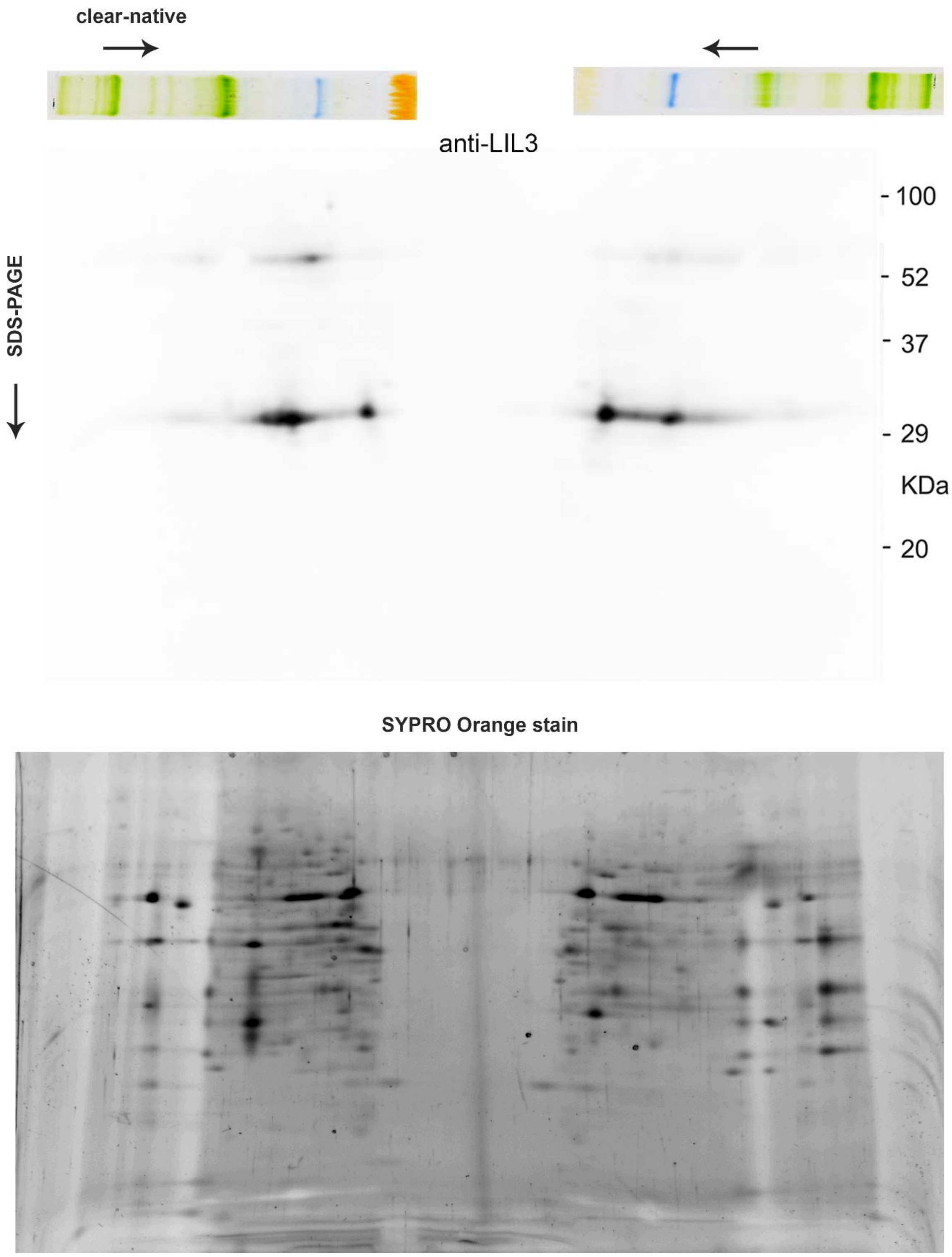
clear-native
anti-LIL3
SDS-PAGE
- 100
- 52
- 37
- 29
KDa
- 20
SYPRO Orange stain

Figure 5c

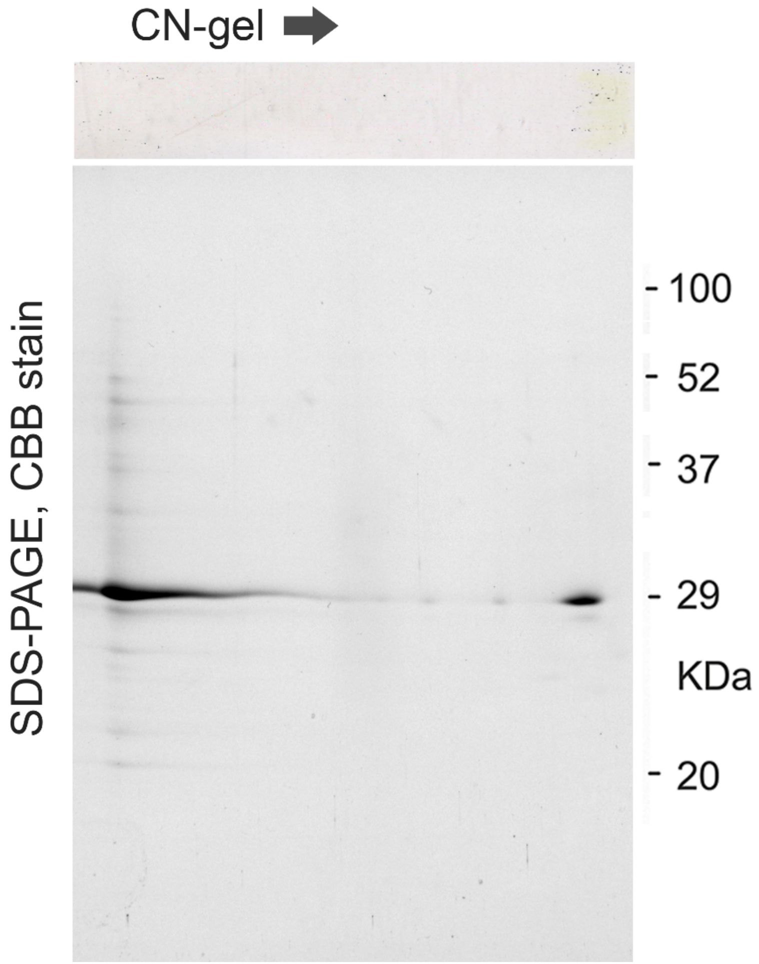

Figure 5e

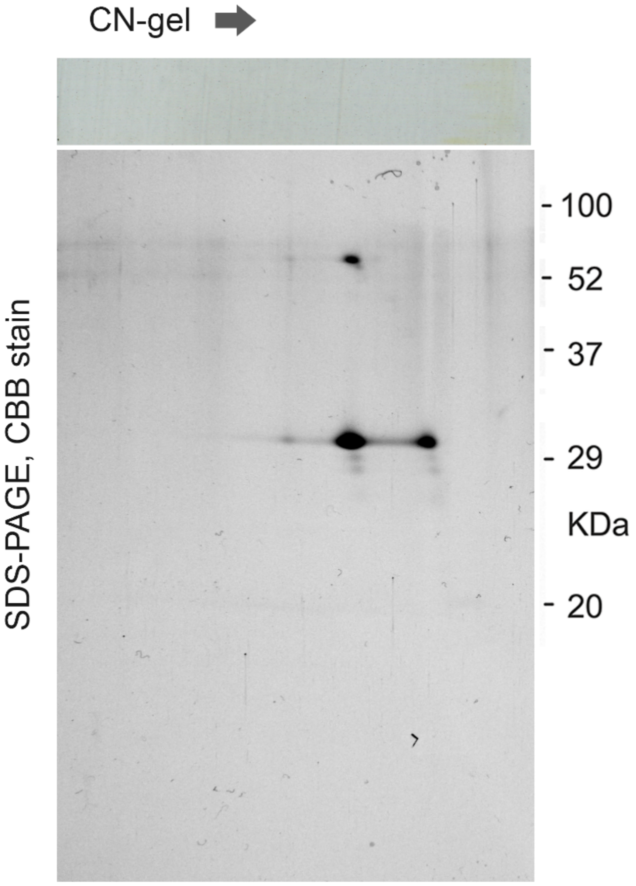

Supplement: Supplementary file 4 — Source Data [file 41467_2021_27155_MOESM4_ESM.zip › source data.pdf]
